# Diabetes Aggravates Post-ischaemic Renal Fibrosis through Persistent Activation of TGF-β_1_ and Shh Signalling

**DOI:** 10.1038/s41598-017-16977-z

**Published:** 2017-12-01

**Authors:** Dong-Jin Kim, Jun Mo Kang, Seon Hwa Park, Hyuk-Kwon Kwon, Seok-Jong Song, Haena Moon, Su-Mi Kim, Jung-Woo Seo, Yu Ho Lee, Yang Gyun Kim, Ju-Young Moon, So-Young Lee, Youngsook Son, Sang-Ho Lee

**Affiliations:** 1 Division of Nephrology, Department of Internal Medicine, Kyung Hee University Hospital at Gangdong, College of Medicine, Kyung Hee University, Seoul, Korea; 2 Department of Genetic Engineering, College of Life Science and Graduate School of Biotechnology, Kyung Hee University Global Campus, Yongin, Korea; 3 Division of Nephrology, Department of Internal Medicine, CHA Bundang Medical Center, CHA University, Seongnam, Korea

## Abstract

Diabetes is a risk factor for acute kidney injury (AKI) and chronic kidney disease (CKD). Diabetic patients are easy to progress to CKD after AKI. Currently, activation of fibrotic signalling including transforming growth factor-β_1_ (TGF-β_1_) is recognized as a key mechanism in CKD. Here, we investigated the influence of diabetes on CKD progression after AKI by using a unilateral renal ischaemia–reperfusion injury (IRI) model in diabetic mice. IRI induced extensive tubular injury, fibrosis and lymphocyte recruitment at 3 weeks after IRI, irrespective of diabetes. However, diabetes showed sustained tubular injury and markedly increased fibrosis and lymphocyte recruitment compared with non-diabetes at 5 week after IRI. The mRNAs and proteins related to TGF-β_1_ and sonic hedgehog (Shh) signalling were significantly higher in diabetic versus non-diabetic IRI kidneys. During the *in vitro* study, the hyperglycaemia induced the activation of TGF-β_1_ and Shh signalling and also increased profibrogenic phenotype change. However, hyperglycaemic control with insulin did not improve the progression of renal fibrosis and the activation of TGF-β_1_ and Shh signalling. In conclusion, diabetes promotes CKD progression of AKI via activation of the TGF-β_1_ and Shh signalling pathways, but insulin treatment was not enough for preventing the progression of renal fibrosis.

## Introduction

After acute kidney injury (AKI), incomplete tubular recovery leads to renal fibrosis and decreased renal function, the common components of chronic kidney disease (CKD)^1^. Generally, in patients with no underlying diseases, recovery from acute kidney injury occurs without significant renal fibrosis. However, transient kidney damage may eventually lead to renal fibrosis in the presence of underlying diseases such as diabetes and CKD^2^. This phenomenon is not only limited to the kidney but can also occur after skin damage or hind limb ischaemia in animal models with diabetes^3,4^. Additionally, the presence of diabetes or underlying CKD are independent risk factors for acute kidney injury after cardiac surgery and coronary/vascular interventions using contrast^5,6^. Therefore, the transition of AKI to CKD is a clinically serious problem for diabetic patients.

Over the past several decades, many studies have been conducted to identify the pathophysiology involved in the development of AKI. However, much remains unknown about the mechanism of transition from AKI to CKD. Recent studies have focused on the role of damaged tubules and a subpopulation of incompletely recovered tubules after AKI, which lead to abnormal growth arrest, failure to redifferentiate into normal tubules, and finally atrophy, as the result of abnormal wound healing^7^. If abnormal wound healing persists or when metabolic derangements impair normal wound healing, atrophic tubules produce persistent and progressively increasing levels of profibrotic signalling molecules such as TGF-β_1_ and Shh^8,9^. These paracrine factors intrinsically play a role in mediating normal wound repair^10,11^. However, persistent activation of these signalling pathways and abnormal cross talk between unhealed tubular cells and interstitial cells such as infiltrating immune cells or activated fibroblasts eventually leads to myofibroblast transformation of pericyte-like fibroblast or bone marrow-derived precursor cells, the final and common pathological feature of renal fibrosis^12,13^.

Both of TGF-β_1_ and Shh pathways are known as typical signalling mediators which lead to renal fibrosis^9,14^. Neutralization of TGF-β_1_ prevents blood vessel loss and development of tubulointerstitial fibrosis after IRI. Additionally, blockage of Shh signalling also reduces renal fibrosis^15,16^. Hyperglycaemia induces high expression levels of TGF-β_1_ and increases levels of Smad 2/3 and CTGF induced by TGF-β_1_^17,18^. However, the relationship between hyperglycaemia and the activation of the Shh pathway is currently unclear.

It is also well known that the mechanism of the injury and repair process is abnormally controlled in the diabetic condition^19^. In addition, aberrant inflammatory cell recruitment and activation of profibrotic signalling pathways are already among the major pathologic mechanisms of diabetic nephropathy^20^.

The unilateral ischaemia reperfusion injury model is suitable for observing the progression of CKD because the characteristics of CKD such as renal mass reduction and tubulointerstitial fibrosis increase with the severity of ischaemic-reperfusion injury^21,22^. Therefore, we hypothesize that enhanced and persistent activation of profibrotic signalling molecules such as TGF-β_1_ and Shh under diabetic conditions induces abnormal fibrotic repair rather than normal wound healing after AKI, which finally accelerates the progression of CKD.

## Results

### Diabetes impaired the improvement of tubular injury and aggravated renal fibrosis after IRI

**Figure 1:**
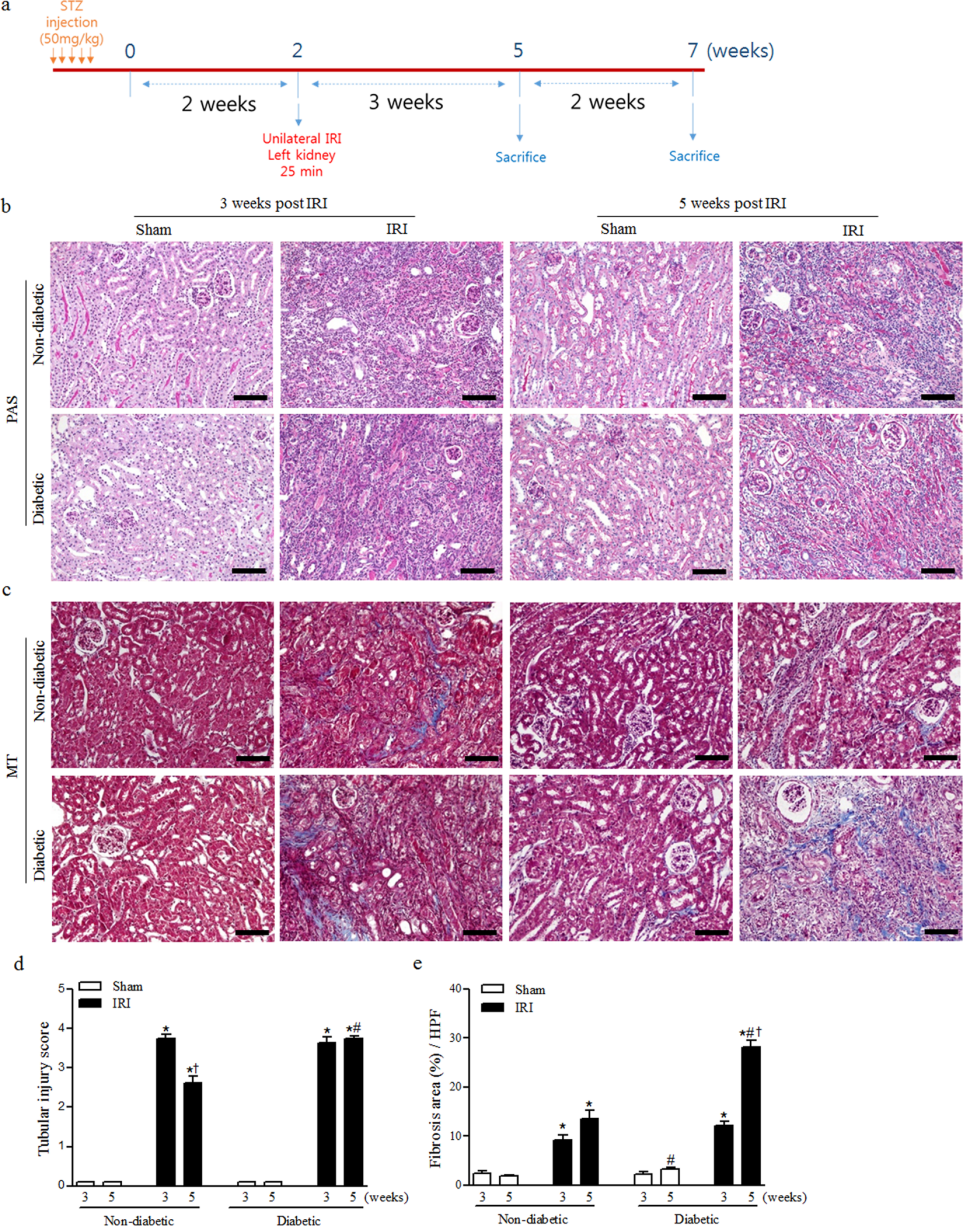
Diabetes impaired the improvement of tubular injury and aggravated renal fibrosis after IRI. (**a**) Experimental design. (**b**) Periodic acid–Schiff (PAS) staining was used to detect interstitial tubular injury at 3 and 5 weeks after IRI. (**c**) Masson’s trichrome (MT) staining was used to detect kidney fibrosis at 3 and 5 weeks after IRI. (**d**) Quantification of interstitial tubular injury based on a grading score at 3 and 5 weeks after IRI. (**e**) Quantification of renal fibrosis at 3 and 5 weeks after IRI. Values are expressed as the mean ± S.E.M. ^*^ *p* < 0.05 versus sham, ^#^ *p* < 0.05 versus non-diabetic, ^†^ *p* < 0.05 versus values at 3 weeks. Scale bar = 80 µm.

To investigate the effect of diabetes on the progression of post-ischaemic renal fibrosis, we used the unilateral renal ischaemia-reperfusion injury (IRI) model in non-diabetic and diabetic mice (Fig. 1a). Compared with sham treatment, IRI induced extensive tubular injury and increased the fibrotic area at 3 weeks after IRI, irrespective of diabetes. While the degree of tubular injury between 3 and 5 weeks after IRI was significantly improved in non-diabetic mice, it was maintained in diabetic mice (Fig. 1b,d). Furthermore, continuous renal mass reduction was seen only in diabetic IRI (Table 1). The degree of renal fibrosis at 5 weeks after IRI was increased in both non-diabetic and diabetic mice. However, its progression was significantly higher in diabetic than non-diabetic mice (Fig. 1c,e). These results indicate that apoptotic tubular damage after IRI lasts longer and is accompanied by the progression of renal fibrosis under diabetic conditions.

**Table 1:** Values are the mean ± S.E.M.

		Non-diabetic		Diabetic	
		Sham	IRI	Sham	IRI
Blood glucose level (mg/dL)	3w	136.0 ± 4.7	131.5 ± 6.4	504.5 ± 42.4^#^	568.7 ± 10.2^#^
	5w	147.3 ± 4.1	157.0 ± 7.3	515.6 ± 49.8^#^	442.4 ± 42.6^#^
Body weight (g)	3w	26.67 ± 1.17	25.08 ± 0.64	22.27 ± 0.87^#^	20.62 ± 0.33^#^
	5w	28.55 ± 0.86	28.30 ± 0.96^†^	24.44 ± 0.81^#^	22.20 ± 1.25^#^
Kidney weight (g)	3w	0.163 ± 0.008	0.107 ± 0.004*	0.168 ± 0.005	0.097 ± 0.006^*^
	5w	0.140 ± 0.007	0.105 ± 0.013	0.162 ± 0.008	0.062 ± 0.005^*#†^
Kidney to body weight ratio (%)	3w	0.613 ± 0.011	0.425 ± 0.014*	0.756 ± 0.028^#^	0.470 ± 0.029^*^
	5w	0.490 ± 0.011^†^	0.380 ± 0.060	0.669 ± 0.051^#^	0.282 ± 0.026^*†^
Kidney length (cm)	3w	1.073 ± 0.021	0.901 ± 0.021*	1.076 ± 0.010	0.844 ± 0.020^*^
	5w	1.073 ± 0.015	0.902 ± 0.041*	1.052 ± 0.011	0.743 ± 0.024^*#†^

^*^ *p* < 0.05 versus Sham, ^#^ *p* < 0.05 versus non-diabetic, ^†^ *p* < 0.05 versus values at 3 weeks.

### Diabetes also aggravated aberrant lymphocyte recruitment after IRI

**Figure 2:**
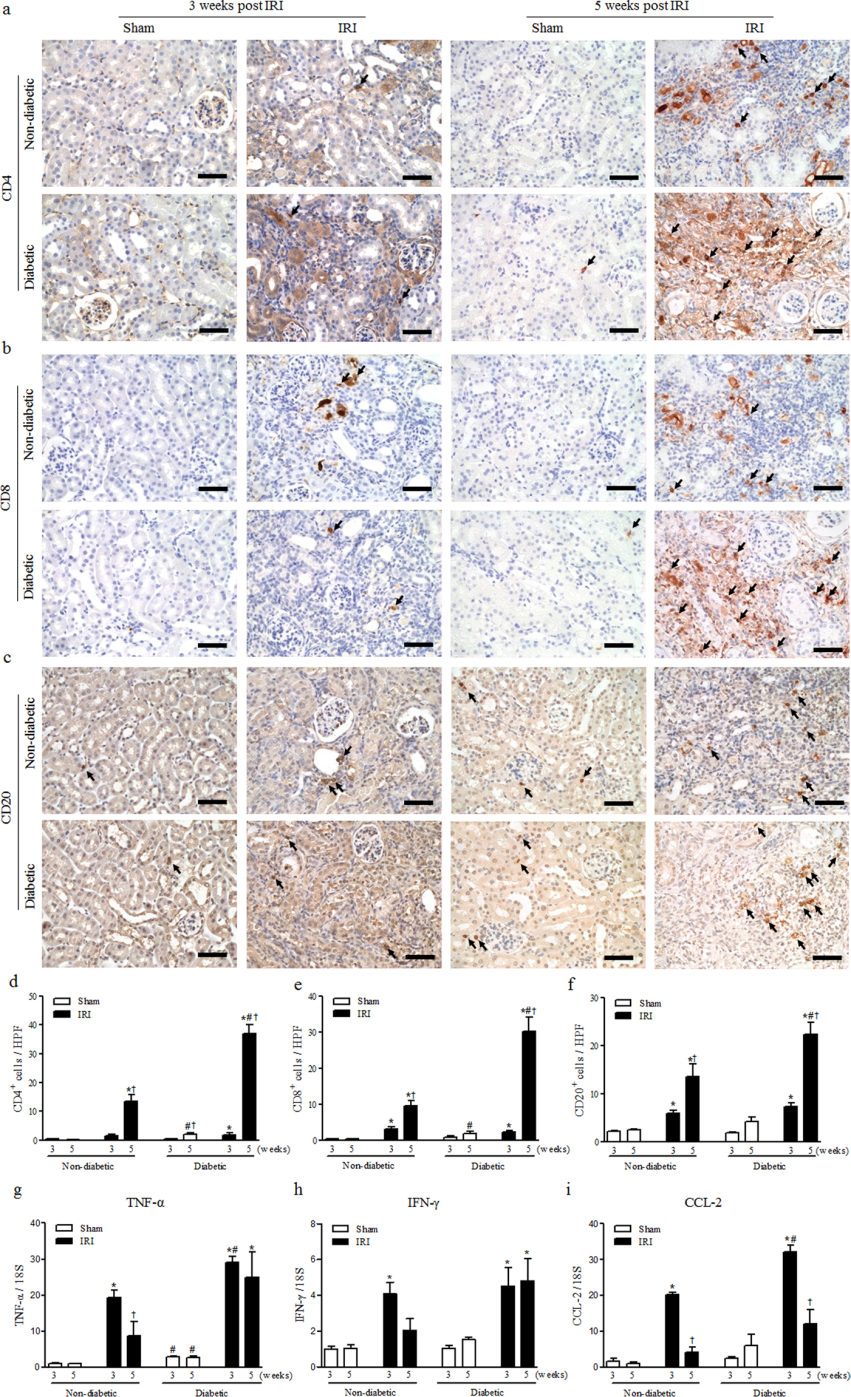
Diabetes aggravated inflammation after IRI. (**a**–**c**) The infiltrated (**a**) CD4-positive, (**b**) CD8-positive T cells and (**c**) CD20-positive B cells measured by immunohistochemistry. (**d**–**f**) Quantification of the number of infiltrated (**d**) CD4-positive, (**e**) CD8-positive T cells and (**f**) CD20-positive B cells in whole kidneys. (**g**–**i**) mRNA expression of inflammatory cytokine markers, (**g**) TNF-α, (**h**) IFN-γ, and (**i**) CCL-2, determined via qRT-PCR. Values are expressed as the mean ± S.E.M. ^*^ *p* < 0.05 versus sham, ^#^ *p* 0.05 versus non-diabetic, ^†^ *p* < 0.05 versus at 3 weeks. Scale bar = 50 µm.

Because aberrant lymphocyte recruitment of is one of the key features of diabetic nephropathy^23^, we assessed the infiltration of CD4^+^, CD8^+^ and CD20^+^ lymphocytes during the progression of kidney fibrosis after AKI. At 3 weeks after IRI, the number of inflammatory immune cells was increased in IRI kidneys, but showed no difference between non-diabetic and diabetic mice. At 5 weeks after IRI, significantly increased infiltration of CD4^+^, CD8^+^ and CD20^+^ lymphocytes was observed in diabetic mice compared to non-diabetic mice (Fig. 2a–f). The mRNA expression levels of inflammatory cytokines including TNF-α, IFN-γ and CCL2 were significantly higher in IRI kidneys than sham kidneys at 3 weeks after IRI (Fig. 2g–i). The expression levels of TNF-α and CCL2 were also higher in diabetic kidneys than in non-diabetic kidneys. From 3 to 5 weeks after IRI, TNF-α and IFN-γ levels were significantly decreased in non-diabetic IRI kidneys, but not in diabetic IRI kidneys (Fig. 2g,h). Although CCL-2 levels were decreased at 5 weeks in both diabetic and non-diabetic kidneys, they were still higher in diabetic compared to non-diabetic IRI kidneys (Fig. 2i). These results indicate that diabetes augmented lymphocyte recruitment to scar kidneys after IRI and maintained the activation of intra-renal inflammation and inflammatory mediators, which could play pivotal roles in the fibrotic cascade.

### Diabetes induced persistent activation of TGF-β_1_ and Shh signalling after IRI

**Figure 3:**
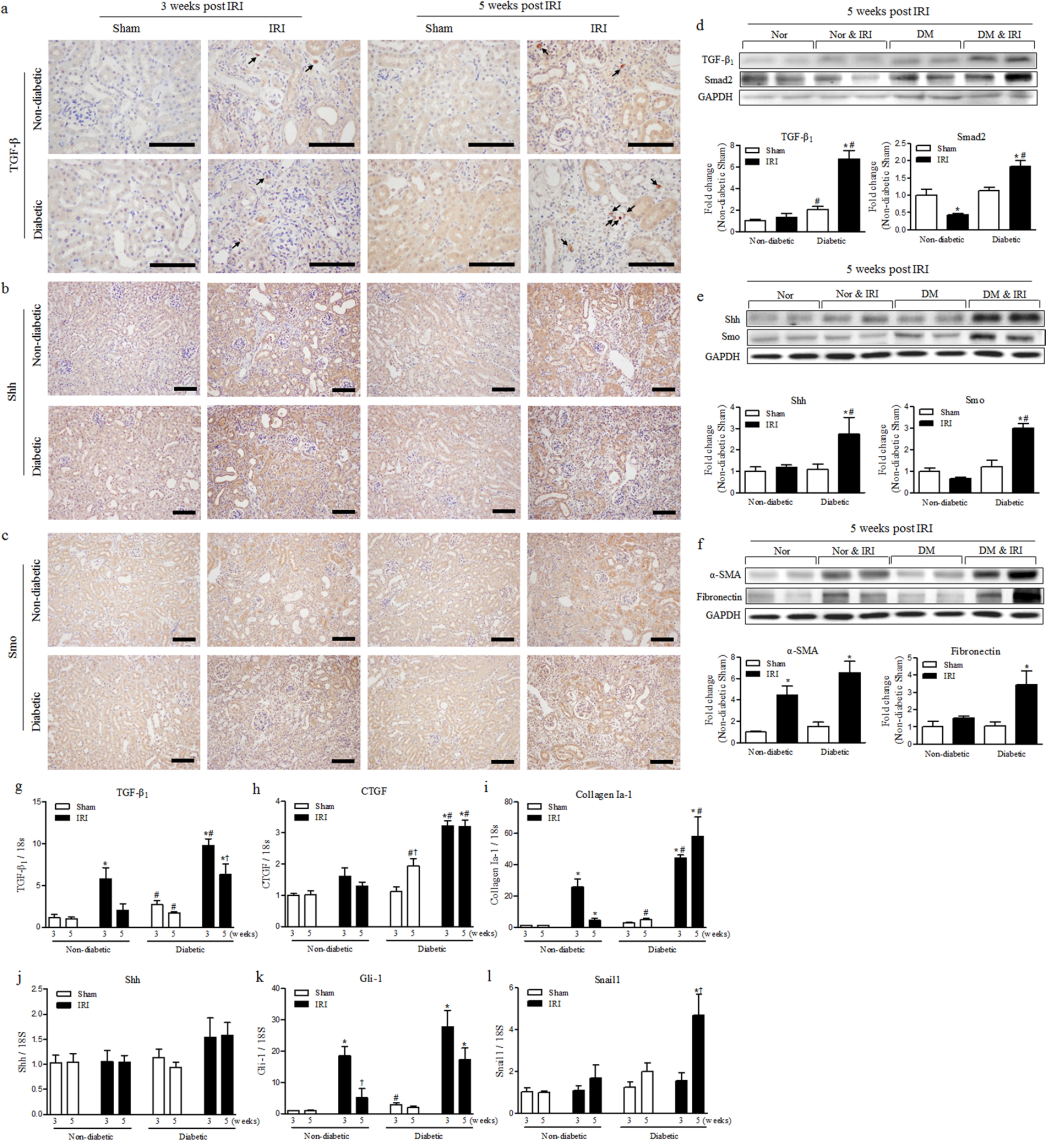
Diabetes induced persistent activation of TGF-β_1_ and Shh signalling. (**a**–**c**) Immunohistochemistry for kidney levels of (**a**) TGF-β_1_, (**b**) Shh and (**c**) Smo at 3 and 5 weeks after IRI. Scale bar = 80 µm. (**d**) The kidney levels of expressed TGF-β_1_ and Smad2 were measured by western blot at 5 weeks after IRI. (**e**) The kidney levels of expressed Shh and Smo were measured by western blot at 5 weeks after IRI. (**f**) The kidney levels of expressed α-SMA and fibronectin were measured by western blot at 5 weeks after IRI. Each protein expression was normalized by GAPDH. The fold-change of each protein was calculated as the ratio of averages versus non-diabetic sham. (**g**–**l**) mRNA expression of the major TGF-β_1_ and Shh signalling pathway factors in the kidney were determined via qRT-PCR at 3 and 5 weeks after IRI. The targets of mRNA were (**g**) TGF-β_1_, (**h**) CTGF, (**i**) collagen I-a1, (**j**) Shh, (**k**) Gli-1 and (**l**) Snail1. Each value of the target mRNA was normalized by 18 S. Values are expressed as the mean ± S.E.M. ^*^ *p* < 0.05 versus sham, ^#^ *p* < 0.05 versus non-diabetic ^†^ *p* < 0.05 versus at 3 weeks.

First, we performed immunohistochemistry for TGF-β_1_, Shh and Smo to identify their expression in kidneys after IRI (Fig. 3a–c). In diabetic kidneys, the slightly increased expression of TGF-β_1_ was shown in remaining tubules and strongly stained the cytoplasm of interstitial cells, especially at 5 weeks after IRI (Fig. 3a). Expression of Shh and Smo was increased in injured tubules rather than the fibrotic area at 3 and 5 weeks after IRI (Fig. 3b,c). The Shh-positive area was slightly reduced in diabetic kidney at 5 weeks because of the replacement of inflammation and expansion of the extracellular matrix. However, Shh expression in remaining and atrophic tubules was increased and more prominent (Fig. 3b). Smo expression was also increased in IRI kidneys, but the increase was higher in diabetic mice at 3 and 5 weeks after IRI (Fig. 3c).

Next, we evaluated the protein expression of TGF-β_1_ and Shh signalling pathways in kidneys at 5 weeks after IRI (Fig. 3d–f). TGF-β_1_ expression was not significantly increased and Smad2 expression was even decreased at 5 weeks after IRI in non-diabetic mice. However, their expression levels were significantly higher in diabetic IRI kidneys compared with sham or non-diabetic kidneys (Fig. 3d). Shh signalling also showed a pattern similar to TGF-β_1_ signalling. Shh expression was increased only in diabetic IRI kidneys at 5 weeks after IRI. Smo expression was also significantly increased only in diabetic kidneys (Fig. 3e). Subsequently, we assessed the expression of α-SMA and fibronectin, markers of fibrosis and extracellular matrix accumulation respectively. α-SMA expression was increased in IRI kidneys compared to sham kidneys, and their tendencies were significantly enhanced in diabetic IRI kidneys. The expression of fibronectin was significantly increased only in diabetic IRI kidneys (Fig. 3f).

Finally, we confirmed the mRNA expression of fibrosis related proteins in kidneys after IRI to demonstrate the effect of diabetes on TGF-β_1_ and Shh signalling pathways. mRNA expression of TGF-β_1_ was induced at 3 weeks after IRI but was more significantly increased in diabetic IRI kidneys. Although TGF-β_1_ mRNA levels were decreased from 3 to 5 weeks after IRI in both groups, they were still significantly higher in diabetic than in non-diabetic IRI kidneys (Fig. 3g). The downstream factors of TGF-β_1_ signalling such as CTGF and their target collagen Ia-1 were increased at 3 weeks after IRI but were significantly higher in diabetic than in non-diabetic IRI kidneys. At 5 weeks after IRI, the mRNA expression levels of CTGF and collagen Ia-1 were slightly decreased in non-diabetic IRI kidneys. However, their expression levels were still maintained or increased in diabetic IRI kidneys (Fig. 3h,i). Although mRNA expression of Shh was not significantly increased in diabetic IRI kidneys, its downstream signal, Gli-1, showed similar expression to TGF-β_1_ (Fig. 3j,k). Its expression was significantly higher in both non-diabetic and diabetic IRI kidneys at 3 weeks. Even during its decrease between 3 and 5 weeks, Gli-1 expression remained significantly higher in diabetic IRI kidneys at 5 weeks (Fig. 3k). Furthermore, the mRNA expression of Snail1, which was known to be a downstream signal of Gli-1, was strongly induced only in diabetic kidneys at 5 weeks after IRI (Fig. 3l). These results showed that TGF-β_1_ and Shh signalling were persistently activated in diabetic IRI kidneys until 5 weeks after IRI, and were associated with increasing renal fibrosis.

### Treatment of insulin was showed no improvement of renal fibrosis after IRI

**Figure 4:**
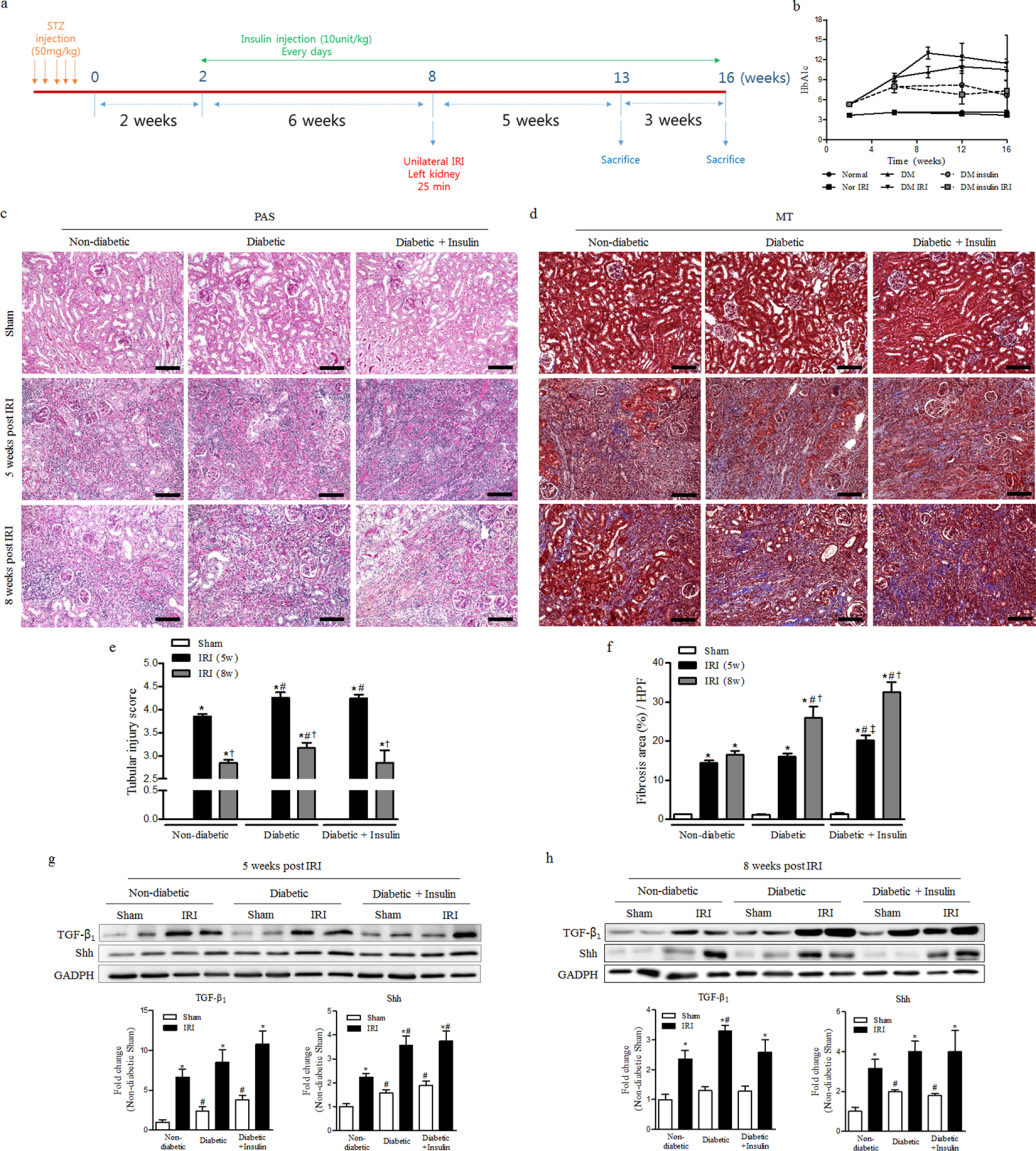
Treatment of insulin was showed no improvement of renal fibrosis after IRI. (**a**) Experimental design. (**b**) The value of HbA1c (**c**) Periodic acid–Schiff (PAS) staining was used to detect kidney tubular injuries and to view necrosis, atrophy and normal tubules at 5 and 8 weeks after IRI. (**d**) Masson’s trichrome (MT) staining was used to detect kidney fibrosis at 5 and 8 weeks after IRI. (**e**) The quantification of total interstitial tubular injury based on a grading score at 5 and 8 weeks after IRI. (**f**) The quantification of renal fibrosis at 5 and 8 weeks after IRI. (**g**) The expression of TGF-b1 and Shh in the kidney at 5 weeks after IRI. (**h**) The expression of TGF-b1 and Shh in the kidney at 8 weeks after IRI. Values are expressed as the mean ± S.E.M. ^*^ *p* < 0.05 versus sham, ^#^ *p* < 0.05 versus non-diabetic, ^†^ *p* < 0.05 versus at 5 weeks, ^‡^ *p* < 0.05 versus between diabetic and diabetic insulin group. Scale bar = 80 µm.

To explore whether the regulation of hyperglycaemia by insulin treatment affect the progression of renal fibrosis after IRI, we conducted follow-up experiments with some modification as shown Fig. 4a. IRI was induced at 8 weeks after streptozotocin injection and insulin was administrated from 2 weeks after the induction of diabetes until the end of the experiment. Insulin administration significantly mitigated the increase of HgA1c levels during the study period (Fig. 4b). From 5 to 8 weeks after IRI, the tubular injury was significantly decreased in all groups. Renal fibrosis was not significantly increased in non-diabetic IRI group. As expected, renal fibrosis in diabetic IRI group was significantly increased at the same time period. However, treatment of insulin to diabetic mice did not affect the tubular injury and renal fibrosis induced by IRI (Fig. 4c–f). Regardless of the treatment of insulin, the protein expressions of TGF-β_1_ and Shh were significantly higher in diabetic IRI groups, not mitigated by insulin. Both TGF-β_1_ and Shh expression were significantly activated in IRI kidneys as compared with those of sham. Shh expression was significantly higher at 5 weeks and TGF-β_1_ expression was significantly higher at 8 weeks in diabetic IRI group as compared with those of non-diabetic IRI group (Fig. 4g,h).

### Hyperglycaemia augmented the effect of Shh and TGF-β_1_ on profibrogenic phenotype change in renal tubular cells

**Figure 5:**
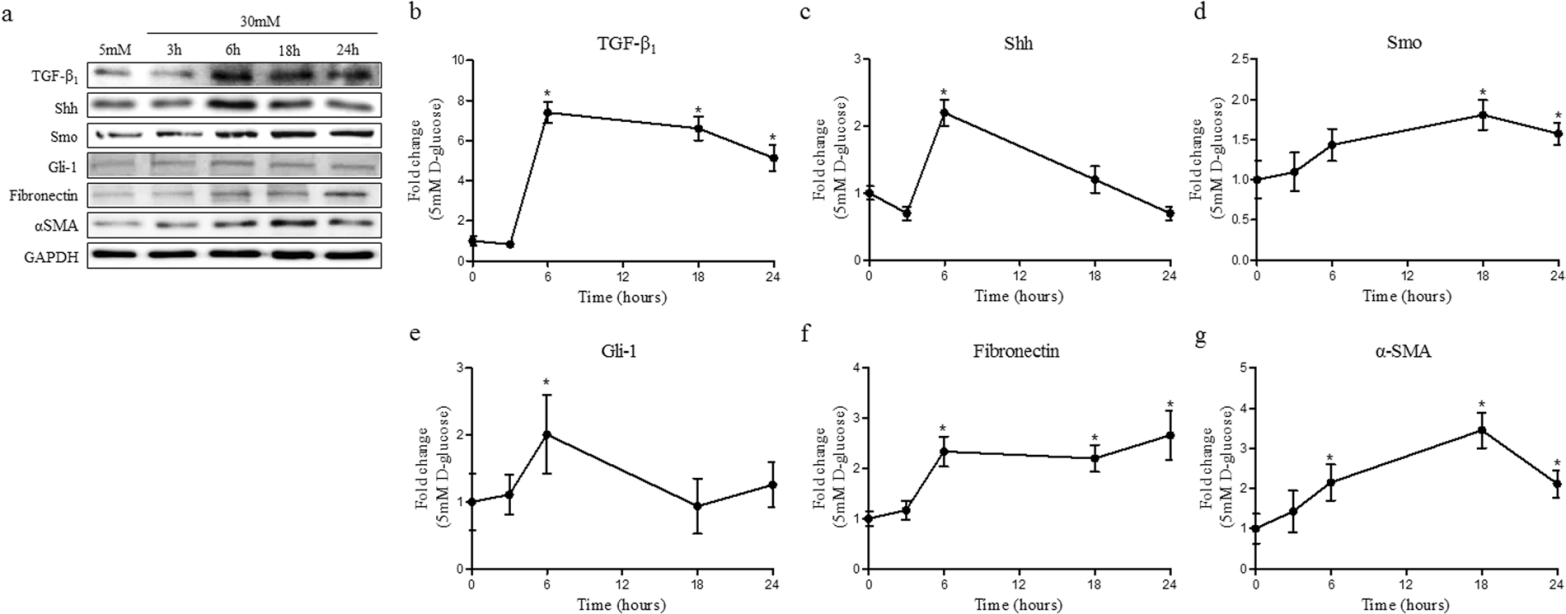
Hyperglycaemia induced EMT via activation of TGF-β_1_ and Shh signalling. HKC-8 cells were cultured in 30 mM D-glucose to induce hyperglycaemic conditions in the cells. (**a**) The expressions of TGF-β_1_, Shh, Smo, Gli-1, fibronectin and α-SMA were measured using western blotting in a time-dependent manner. The fold-changes of (**b**) TGF-β_1_, (**c**) Shh, (**d**) Smo, (**e**) Gli-1, (**f**) fibronectin and (**g**) α-SMA were calculated as the ratios of averages versus a 5 mM D-glucose control. Expression of each protein was normalized by GAPDH. Values are expressed as the mean ± S.E.M. ^*^ *p* < 0.05 versus 5 mM D-glucose control.

To demonstrate the effect of hyperglycaemia on TGF-β_1_ and Shh signalling, HKC-8 was cultured under hyperglycaemic conditions. Compared to normoglycaemic conditions (5 mM D-Glucose, NG), the hyperglycaemic state (30 mM D-Glucose, HG) induced TGF-β_1_ and Shh expression at 6 hours. Although Shh expression was significantly reduced at 24 hours, TGF-β_1_ was maintained until 24 hours (Fig. 5a–c). Smo expression was significantly increased in HG at 18 and 24 hours (Fig. 5d). However, Gli-1 was induced at 6 hours and returned to its basal level at 18 hours (Fig. 5e). Fibronectin and α-SMA expression were induced at 6 hours and were maintained through 24 hours (Fig. 5f,g). These results suggest that hyperglycaemia activates the profibrotic signalling pathway through the activation of Shh as well as TGF-β_1_.

**Figure 6:**
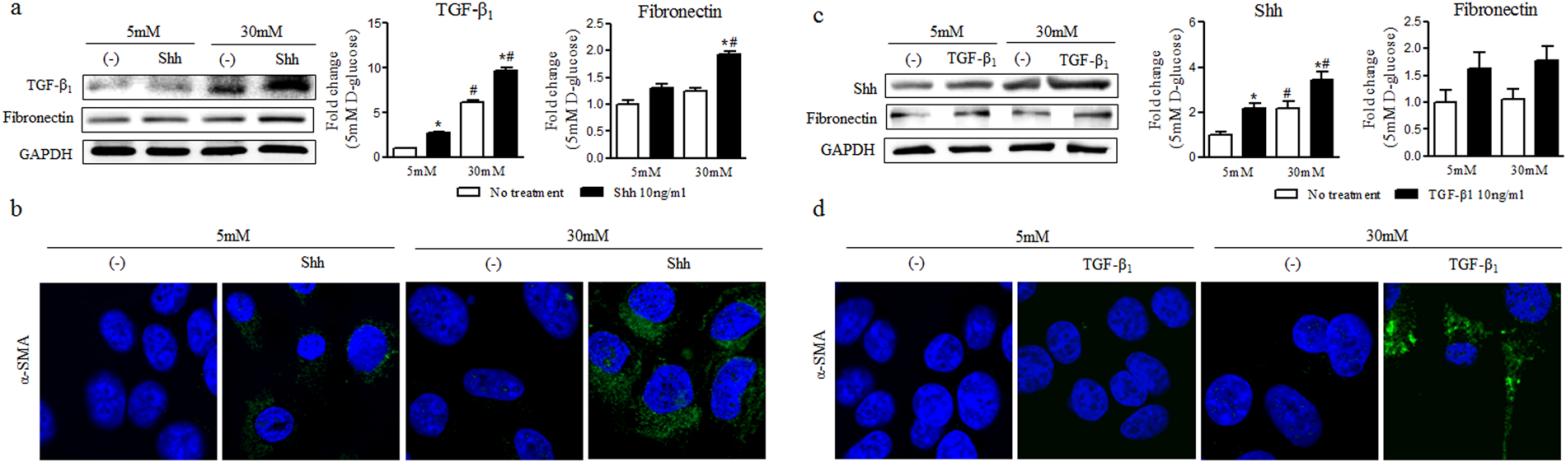
Hyperglycaemia augmented the effect of Shh and TGF-β_1_ on profibrogenic phenotype change in renal tubular cells. HKC-8 cells were cultured in 5 mM D-glucose or 30 mM D-glucose with 10 ng/ml Shh or 10 ng/ml TGF-β_1_. (**a**) Expression levels of TGF-β_1_ and fibronectin at 24 hours after Shh treatment. (**b**) Representative confocal fluorescence images of α-SMA were examined at 24 hours after Shh treatment. (**c**) Expression levels of Shh and fibronectin at 24 hours after TGF-β_1_ treatment. (**d**) Representative confocal fluorescence images of α-SMA were examined at 24 hours after TGF-β_1_ treatment. Expression of each protein was normalized by GAPDH. The fold-change of each protein was calculated as the ratio of averages versus a 5 mM D-glucose control. Values are expressed as the mean ± S.E.M. ^*^ *p* < 0.05 versus 5 mM D-glucose or 30 mM D-glucose controls, ^#^ *p* < 0.05 versus 5 mM D-glucose with treatment.

Next, we exposed recombinant human Shh or TGF-β_1_ to NG or HG for 24 hours. Shh treatment increased the expression of TGF-β_1_ in both NG and HG (Fig. 6a). Expression levels of α-SMA and fibronectin were increased in both NG and HG by Shh treatment after 24 hours. However, the induction of α-SMA and fibronectin by Shh was significantly greater in HG than in NG (Fig. 6a,b). In the case of TGF-β_1_ treatment, Shh expression was increased in both NG and HG (Fig. 6c). Fibronectin expression was increased in both NG and HG at 24 hours after TGF-β_1_ treatment. However, there was no difference between NG and HG (Fig. 6c). α-SMA expression of was increased by TGF-β_1_ after 24 hours in HG, but not in NG (Fig. 6d). These results indicate that hyperglycaemia increased the expression of Shh or TGF-β_1_ in terms of profibrogenic phenotype change in HKC-8 cells. Although Shh and TGF-β_1_ interacted with each other, hyperglycaemia did not affected the crosstalk between Shh and TGF-β_1_.

### Insulin increased the expressions of Shh and TGF-β_1_ without affecting the interaction between Shh and TGF-β1 in hyperglycaemic conditions

**Figure 7:**
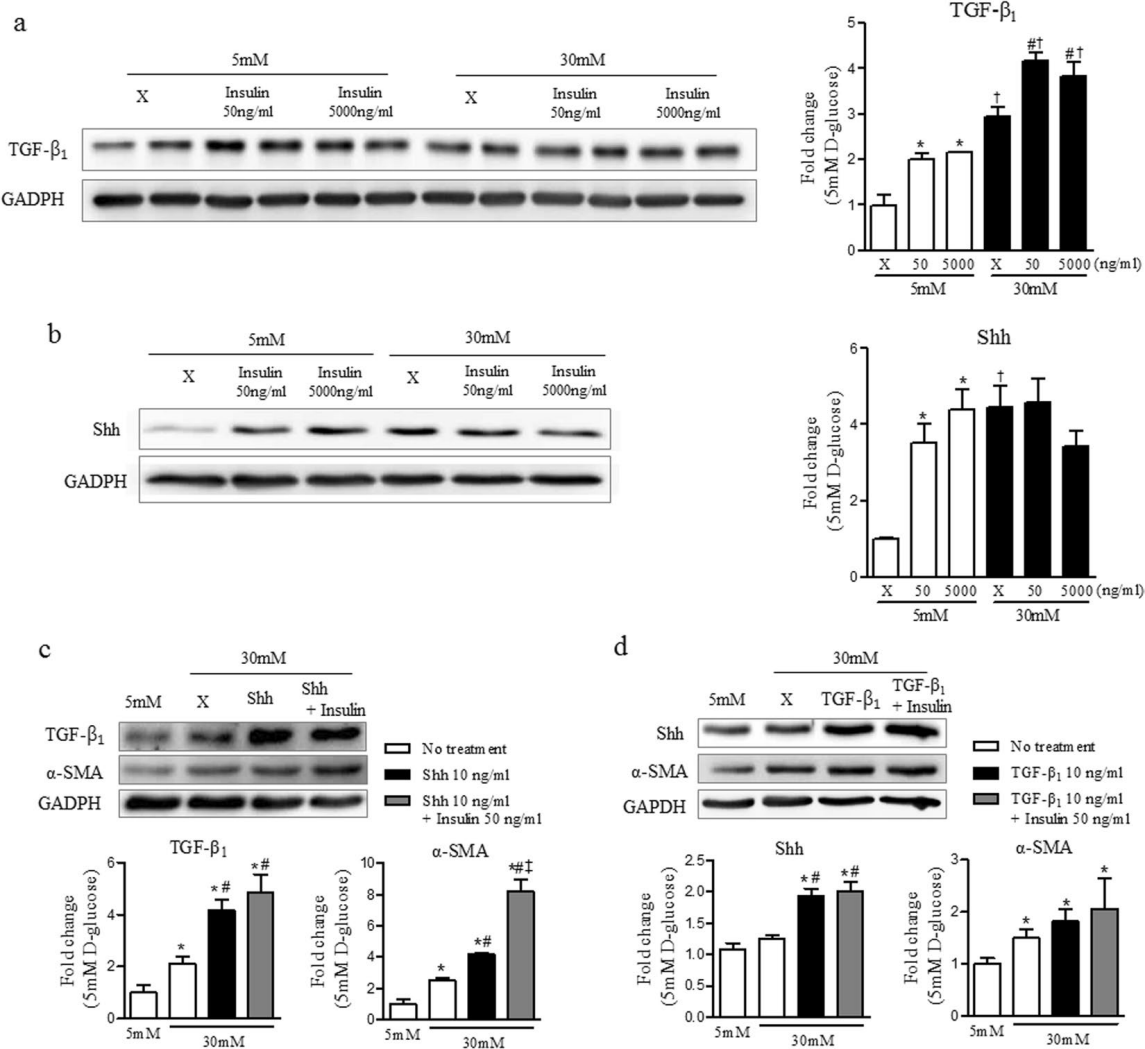
Insulin increased the expressions of Shh and TGF-β_1_ without affecting the interaction between Shh and TGF-β_1_ in hyperglycaemic condition. (**a**) Expression level of TGF-β_1_ at 24 hours after insulin treatment. (**b**) Expression level of Shh at 24 hours after insulin treatment. (**c**) Expression levels of TGF-β_1_ and α-SMA at 24 hours after insulin and Shh treatment. (**d**) Expression levels of Shh and α-SMA at 24 hours after insulin and TGF-β_1_ treatment. Expression of each protein was normalized by GAPDH. The fold-change of each protein was calculated as the ratio of averages versus a 5 mM D-glucose control. Values are expressed as the mean ± S.E.M. ^*^ *p* < 0.05 versus 5 mM D-glucose, ^#^ *p* < 0.05 versus 30 mM D-glucose. ^†^ *p* < 0.05 versus between 5 mM D-glucose with insulin treatment and 30 mM D-glucose with insulin treatment. ^‡^ *p* < 0.05 versus 30 mM D-glucose with Shh or TGF-β_1_ treatment.

To investigate the mechanisms by which insulin has no effect on renal fibrosis after IRI, HKC-8 was cultured in LG and HG with insulin stimulation. The expressions of TGF-β_1_ and Shh were increased by insulin stimulation as well as in HG. In HG, the expression of Shh was not enhanced by insulin, but the expression of TGF-β_1_ was significantly increased by insulin (Fig. 7a,b). The increased mutual stimulation between Shh and TGF-β_1_ under hyperglycaemic condition was not altered by insulin (Fig. 7c,d). Insulin also did not mitigate the activated expression of α-SMA by Shh or TGF-β_1_ under the HG, but significantly increased the Shh-induced expression of α-SMA in HG (Fig. 7c).

## Discussion

Among diverse causes of chronic kidney disease, diabetes accounts for the largest portion of kidney failure, accounting for approximately 50% of cases in the developed world^24^. High blood pressure, poor glycaemic control and albuminuria are well-known risk factors for the development or progression of diabetic nephropathy. However, these factors cannot explain all of the interindividual variability in the rate of progression to end-stage of CKD^25^. Recently, AKI episodes have been known to be associated with a cumulative risk for developing advanced CKD in diabetes mellitus, independent of other major risk factors of progression^26^. Additional loss of parenchyma caused by failed repair of AKI has been implicated in the development and progression of experimental nephropathy in diabetic rodents using an acute-on-chronic injury model^7^. However, the underlying mechanism of the progression of CKD after AKI under diabetic conditions is not currently well known. Here, we demonstrated why diabetes is particularly prone to progression to CKD after AKI through three major findings in our study.

First, diabetes exacerbated abnormal lymphocyte infiltration after IRI. In this study, the recruitment of CD4^+^, CD8^+^ and CD20^+^ lymphocytes in the kidney was increased at 3 weeks after IRI. However, there was no difference between non-diabetic and diabetic kidneys at 3 weeks after IRI. At 5 weeks after IRI, levels of infiltrating CD4^+^, CD8^+^ and CD20^+^ lymphocytes were sharply increased, but were significantly higher in diabetic IRI kidneys than in non-diabetic IRI kidneys. Aberrant recruitment of T and B lymphocytes is one of the major findings in diabetic nephropathy^27,28^. At the early phase of AKI, the major infiltrating immune cells in the kidney are neutrophils and macrophages. In contrast, the recruitment of lymphocytes into the injured kidney was reported to be prominent during the recovery phase^29,30^. The exact role of these lately infiltrating lymphocytes has not been studied extensively. However, some studies suggested that B cell infiltration after AKI might hinder normal wound healing^31^. Interestingly, recent studies also suggested that infiltrated lymphocytes directly affected renal fibrosis by increasing levels of TGF-β_1_^32,33^.

The major finding of this study was that diabetes induced persistent activation of TGF-β_1_ and Shh signalling. In this study, persistent activation of the TGF-β_1_ and Shh signalling pathways was maintained only in the diabetic group at 5 weeks after IRI. Furthermore, the downstream signalling molecules Smo and Gli-1 were also persistently enhanced in diabetic IRI kidneys. These results showed that TGF-β_1_ and Shh signalling were persistently activated in diabetic IRI kidneys until 5 weeks after IRI as well as were associated with increasing renal fibrosis. The TGF-β_1_ signalling pathway reduces tissue injury and promotes tubule restoration through epithelial de-differentiation at the early stage of acute injury^34,35^. However, under maladaptive wound healing conditions, TGF-β_1_ signalling could promote abnormal organ fibrosis through myofibroblast differentiation from fibroblasts or bone marrow-derived precursor cells^36,37^. The Shh signalling pathway could also modulate the activation of myofibroblast and kidney fibrosis after tubular injury^38–40^. Shh was secreted from the damaged renal tubular epithelium and induced fibroblasts to differentiate into myofibroblasts^41,42^. Furthermore, the overexpression of Shh was reported to increase extracellular matrix (ECM) synthesis^40^. Contrary to the TGF-β_1_ signalling pathway, the effect of diabetes on Shh signalling pathway has not yet been clarified. However, the results of this study indicate that sustained tissue injury, activation of inflammation as well as hyperglycaemia itself could be suggested as mechanisms underlying the activation of Shh signalling during CKD progression after diabetic AKI.

We also demonstrated using *in vitro* experiments that hyperglycaemia induced the activation of Shh signalling as well as TGF-β_1_ and augmented the effect of both Shh and TGF-β_1_ on profibrogenic phenotype change in renal tubular cells. Although cell culture models in diabetic kidney injuries such as our study, have several limitations in mimicking *in vivo* tissue environment, these results could be explained by the finding that hyperglycaemia increased the activation of Shh and TGF-β_1_. Recent studies indicated that Shh signalling could be activated by TGF-β_1_^43^. Furthermore, TGF-β_1_ and its receptor were also reported to be increased by the activation of Shh signalling^43^. The crosstalk between Shh and TGF-β_1_ was also recently reported to enhance kidney fibrosis in several models of fibrotic kidney diseases^44–46^. Our results also showed the mutual influence of Shh and TGF-β_1._ However, hyperglycaemia did not have a significant effect on the interaction between Shh and TGF-β_1._

Since insulin treatment is one way to control the hyperglycemia, which may be the target to rescue the kidney fibrosis following IRI, insulin treatment could be expected to reduce the expression of TGF-β_1_ and Shh signaling and finally ameliorate the progression of renal fibrosis after IRI.

To answer this question, we conducted the additional experiment with some modifications as in Fig. 4. As expected, longer-lasting diabetes before IRI induction and hyperglycemia extended to 8 weeks significantly aggravated the post-ischaemic fibrosis. However, treatment of insulin did not improved renal fibrosis in our animal model showing progressive kidney fibrosis after IRI, even though insulin showed significantly improved control of blood glucose level. Our results also showed that treatment of insulin had no effect on the expression of TGF-β_1_ and Shh at both 5 and 8 weeks after IRI. Negative result with insulin treatment could be anticipated, because the correction of hyperglycemia might be suboptimal in our experiment (mean HgA1c 6.5%) or ischaemic kidney damage in diabetic group was too severe to overcome with the blood glucose control. However, we had noted the results in the previous reports, which showed that insulin induced the expression of TGF-β_1_ in renal proximal tubular cells^47^, and insulin stimulated SLT2-mediated tubular glucose absorption via oxidative stress generation^48^.

In our *in vitro* experiment, we also confirmed that insulin itself could induce the expression of TGF-β_1_ and also affect the expression of Shh in HKC-8 cells. However, insulin was not altered the mutual interaction between Shh and TGF-β_1_ under high glucose condition. Based on these results, control of hyperglycemia by treatment of insulin may not be enough for preventing renal fibrosis after IRI, and further studies on the effect of other hypoglycemic agents with different action mechanism on tubular cells, are necessary.

In conclusion, we demonstrated that diabetes promoted CKD progression following AKI through recruitment of lymphocytes and persistent activation of TGF-β_1_ and Shh signalling pathways. The control of hyperglycaemia with insulin administration was not enough for preventing the progression of renal fibrosis. Future study of the mechanism of renal fibrosis progression after diabetic AKI will contribute to solving the unresolved mechanisms underlying kidney fibrosis and to locating potential therapeutic targets for treatment of diabetic kidney failure.

## Materials and Methods

### Animals and experimental design

#### Effect of hyperglycaemia on the progression of renal fibrosis after IRI

Diabetes was induced by streptozotocin (Sigma-Aldrich, St. Louis, MO, USA) injected once a day at a concentration of 50 mg/kg for five consecutive days into 8-week-old male C57BL/6 mice. After 2 weeks, a unilateral renal ischaemia–reperfusion injury (IRI) model was established in non-diabetic mice and diabetic mice with blood glucose levels above 250 mg/dL. We induced ischaemia in the left kidneys of the mice using a clamp to obstruct blood circulation for 25 minutes. During the ischaemic period, mice were maintained within a body temperature range of 36–37 °C using a heating pad and a rectal temperature probe. Mice were sacrificed at 3 or 5 weeks after IRI and divided into four groups: (1) non-diabetic controls (Nor Sham), (2) non-diabetic IRI (Nor & IRI), (3) diabetic controls (DM sham), and (4) diabetic IRI (DM & IRI). Mice were monitored for body weights and blood glucose levels every 2 weeks and HbA1c levels at 2 and 6 weeks. All animal experiments were performed in compliance with the guidelines of the Animal Research Ethics Committee of Kyung Hee University and Institutional Animal Care and Use committee Kyung Hee University Hospital at Gangdong, Seoul, Korea (approval number: KHNMC AP 2015-001).

#### Effect of insulin treatment on the progression of post-ischaemic renal fibrosis

At 2 weeks after diabetic induction, Insulin (10 unit/kg, insulin glargine, LANTUS® by Sanofi-Aventis was administrated once a day until the end of the experiment. At 8 weeks after diabetic induction, the unilateral IRI was established in non-diabetic, diabetic and insulin-treated diabetic mice. Mice were sacrificed at 5 and 8 weeks after IRI and divided into six groups: (1) non-diabetic controls (Nor Sham), (2) non-diabetic IRI (Nor & IRI), (3) diabetic controls (DM Sham), (4) diabetic IRI (DM & IRI), (5) insulin-treated diabetic controls (Ins Sham) and (6) insulin-treated diabetic IRI (Ins & IRI). Blood glucose level was checked every 2 weeks and HbA1c was checked at 2, 6, 9 and 12 weeks after diabetic induction. All animal experiments were performed in compliance with the guidelines of the Animal Research Ethics Committee of Kyung Hee University and Institutional Animal Care and Use committee Kyung Hee University Hospital at Gangdong, Seoul, Korea (approval number: KHNMC AP 2016-007).

### Histology

Sections were cut at 4 µm thickness. For the histological assessment of tubulointerstitial injury (tubular necrosis, tubular atrophy, and glomerular cysts), the sections were stained with periodic acid-Schiff reagent. Ten corticomedullary fields were examined in each section at 200x magnification, and a semiquantitative analysis of tubulointerstitial injury was performed. Total tubular injury was graded on a scale of 0 to 5 based on the percentage of normal tubules and the amount of tubular necrosis and tubular atrophy as follows: 0, absent; 1, 1–25%; 2, 26–50%; 3, 51–75%; 4, 76–99%; and 5, 100%. Fibrosis was quantified using Masson’s trichrome staining and computer-assisted image analysis. For immunohistochemistry, we used the Bond Polymer Refine Detection system (Vision BioSystems, Hingham, MA, USA) with antibodies against CD4, CD8 and CD20. Four-micron-thick kidney sections were deparaffinized using Bond Dewax solution, and an antigen retrieval procedure was then performed using Bond ER solution for 30 minutes at 100 °C. Endogenous peroxidase activity was terminated by incubating the tissues with hydrogen peroxide for 5 minutes. The sections were then incubated with CD4 (1:100, Abcam, Cambridge, MA, USA), CD8, and CD20 (1:100; Abcam, Cambridge, MA, USA) antibodies using a biotin-free polymeric horseradish peroxidase-linked antibody conjugate system (Vision BioSystems, Hingham, MA, USA). To assess T and B lymphocyte infiltration, CD4^+^/CD8^+^/CD20^+^ cells were counted in 20 randomly selected fields from each section at 400x magnification under a light microscope.

### ***In vitro*** cell culture

The human renal proximal tubular epithelial cell line HKC8 was obtained from Dr. L. Rausen (Johns Hopkins University, Baltimore, MD, USA) and was maintained in Dulbecco’s Modified Eagle’s Medium supplemented with Ham’s F12 medium (DMEM/F12; Invitrogen, Carlsbad, CA, USA) DMEM/F12 was supplemented with 5% foetal bovine serum and 1% penicillin/streptomycin (WelGENE, Daegu, Korea). Before experiments, cells were maintained in a medium containing 5 mM D-glucose. 3 × 10^5^ cells were seeded in a 60 mm dish with 5 mM D-glucose and cultured for 24 hours^49^. To confirm the effect of hyperglycaemia on the profibrogenic signalling pathway in a time-dependent manner, we changed the media containing 30 mM D-glucose and collected cells at 3, 6, 18 and 24 hours after treatment. To demonstrate the effects of TGF-β_1_ and Shh on HKC-8 cells, media were changed following these conditions: (1) 5 mM D-glucose, (2) 5 mM D-glucose + TGF-β_1_ 10 ng/ml, (3) 5 mM D-glucose + Shh 10 ng/ml, (4) 30 mM D-glucose, (5) 30 mM D-glucose + TGF-β_1_ 10 ng/ml, and (6) 30 mM D-glucose + Shh 10 ng/ml. To demonstrate the effect of insulin on the expressions of TGF-β_1_ and Shh in HKC-8 cells, media were changed following these conditions: (1) 5 mM D-glucose, (2) 5 mM D-glucose + insulin 50 ng/ml, (3) 5 mM D-glucose + insulin 5000 ng/ml, (4) 30 mM D-glucose, (5) 30 mM D-glucose + insulin 50 ng/ml, and (6) 30 mM D-glucose + insulin 5000 ng/ml. To identify the effect of insulin on the sensitivity to TGF-β_1_ and Shh, media were changed following these conditions: (1) 5 mM D-glucose, (2) 30 mM D-glucose, (3) 30 mM D-glucose + TGF-β_1_ 10 ng/ml, (4) 30 mM D-glucose + TGF-β_1_ 10 ng/ml + insulin 50 ng/ml, (5) 30 mM D-glucose + Shh 10 ng/ml, and (6) 30 mM D-glucose + Shh 10 ng/ml + insulin 50 ng/ml. After 24 hours, cells were collected for protein analysis.

### Isolation of total RNA and real-time PCR

Total RNA was extracted from kidney tissue by using the Nucleospin® RNA kit (Macherey-Nagel, Düren, W. Germany) according to the manufacturer’s instructions and was quantified using a NanoDrop 2000 Spectrophotometer (Thermo Fisher Scientific Inc., San Jose, CA, USA). Complementary DNA was synthesized using random primers (Promega, Madison, WI, USA), DNTP mixture (TaKaRa Bio Inc., Otsu-shi, Shiga, Japan) and M-MLV reverse transcriptase (Mbiotech Inc., Hanam, Korea). Real-time PCR was performed in reactions with a final volume of 20 μl containing 1 μl of cDNA, 10 pmol of each sense and antisense primer, and 17 μl of Power SYBR® Green PCR Master mix (Applied Biosystems, Beverly, MA, USA) and was detected using the Applied Biosystems® StepOnePlus™ Real-Time PCR System. The primers for TGF-β_1_, CTGF, collagen IV-a1, collagen I-a1, Snail1, Shh, Gli-1, IFN-γ, TNF-α, CCl-2 and 18 S were purchased from Mbiotech. Each sample was run in duplicate in separate tubes to quantify target gene expression, and the results were normalized to 18 S expression.

### Western blot analysis

Cells and kidney tissues were washed with PBS and lysed in the M-PER mammalian protein extraction reagent with protease inhibitor cocktail (Thermo Fisher Scientific Inc., San Jose, CA, USA). Proteins were separated with 8–15% SDS-PAGE and then were transferred onto a nitrocellulose membrane (Millipore, Madrid, Spain) by electroblotting. The membrane was blocked for 1 hour at room temperature and then was incubated overnight at 4 °C with anti-Shh, anti-E-cadherin, anti-Smad2, anti-Smo, anti-Gli-1 (1:1000, Santa Cruz biotechnology, Santa Cruz, CA, USA), anti-fibronectin (R&D system Inc. Minneapolis, MN, USA), anti-Bax, anti-Bcl-2, anti-TGF-β_1_ (1:1000, Cell Signaling Technology, Beverly, MA, USA), and α-SMA (1:1000 Abcam Inc. Cambridge, MA, USA) primary antibodies. Subsequently, the membranes were stained with horseradish peroxidase-conjugated goat anti-rabbit or mouse immunoglobulin G (1:2,000, Santa Cruz biotechnology, Santa Cruz, CA, USA). The immunoreactive bands were detected by chemiluminescence (enhanced chemiluminescence; BioFX Laboratories Inc., Owings Mills, Maryland, USA). GAPDH (1:2,000, Santa Cruz biotechnology, Santa Cruz, CA, USA) was used as an internal control.

### Confocal microscopy

Cells were fixed with 4% paraformaldehyde, permeabilized with 0.2% Triton X-100 solution, blocked with bovine serum albumin (BSA), and incubated with primary antibodies for 2 hours. After washing with PBS, the samples were re-incubated with secondary antibodies conjugated with Alexa Fluor 488 or Texas Red (Life Technologies, Gaithersburg, MD, USA) for 1 hour. Cells were counterstained with DAPI to delineate the nuclei, and the sections were examined using confocal microscopy (LSM-700; Carl Zeiss, Jena, Thuringia, Germany).

### Statistical analyses

All values are expressed as the mean ± SE. The results were analysed using the Kruskal-Wallis nonparametric test for multiple comparisons. Significant differences detected using the Kruskal-Wallis test was confirmed using the Wilcoxon rank sum and Mann-Whitney tests (to compare mean differences). *p*-values < 0.05 were considered to indicate statistical significance.

## Electronic supplementary material

Supplementary Information
