# Supplementary material for: Diabetes Aggravates Post-ischaemic Renal Fibrosis through Persistent Activation of TGF-β1 and Shh Signalling
Source: Sci Rep. 2017 Dec 1;7:16782. doi: 10.1038/s41598-017-16977-z (PMC5711892; doi:10.1038/s41598-017-16977-z)

Supplementary information.

Diabetes Aggravates Post-ischaemic Renal Fibrosis through Persistent Activation of TGF-β_1 and Shh Signalling.

Dong-Jin Kim[1,2], Jun Mo Kang[3], Seon Hwa Park[1], Hyuk-Kwon Kwon[1], Seok-Jong Song[1], Haena Moon[1], Su-Mi Kim[1], Jung-Woo Seo[1], Yu Ho Lee[1], Yang Gyun Kim[1], Ju-Young Moon[1], So-Young Lee[3], Youngsook Son[2,*], Sang-Ho Lee[1,*]

[1]Division of Nephrology, Department of Internal Medicine, Kyung Hee University Hospital at Gangdong, College of Medicine, Kyung Hee University, Seoul, Korea

[2]Department of Genetic Engineering, College of Life Science and Graduate School of Biotechnology, Kyung Hee University Global Campus, Yongin, Korea

[3]Division of Nephrology, Department of Internal Medicine, CHA Bundang Medical Center, CHA University, Seongnam, Korea

[*]Correspondence:

Prof. Sang-Ho Lee

Division of Nephrology, Department of Internal Medicine, Kyung Hee University Hospital at Gangdong, College of Medicine, Kyung Hee University, Dongnam-ro 892, Gangdong-gu, Seoul, 05278, Republic of Korea.

Phone: +82-2-440-6121; Fax: +82-2-440-8150; E-mail: lshkidney@khu.ac.kr

Prof. Youngsook Son

Graduate School of Biotechnology & Department of Genetic Engineering,

College of Life Science, Kyung Hee University Global Campus, Deogyeong-daero 1732, Giheung-gu, Yongin, 17104, Republic of Korea.

Phone: +82-31-201-3829; Fax: +82-31-206-3829; E-mail: ysson@khu.ac.kr

Supplementary Fig. S1. (a) Masson's trichrome (MT) staining was used to detect kidney fibrosis after IRI. Diabetic sham kidney was collected at 16 weeks after STZ injection and diabetic IRI contralateral kidney was collected at 8 weeks after IRI (at 16 weeks after STZ injection). (b) Quantification of renal fibrosis in normal sham, diabetic sham and diabetic IRI contralateral kidney. All of them were not different in renal fibrosis.

Supplementary Fig. S2. HKC-8 cells were cultured in 30 mM D-glucose to induce hyperglycaemic conditions in the cells and in 5 mM D-glucose + 25 mM Mannitol to be used as an osmotic control. (a) The expression of TGF-β_1 was measured by western blotting at 24 hours after treatment. (b) The fold-change of TGF-β_1 was calculated as the ratios of averages versus a 5 mM D-glucose control. Expression of TGF-β_1 was normalized by GAPDH. Values are expressed as the mean ± S.E.M. $^{*}p < 0.05$ versus 5 mM D-glucose control. The original images of (c) TGF-β_1 and (d) GAPDH

Supplementary Fig. S3. The original image of Fig. 3d-f. The original images of Fig. 3d were (a) TGF-β_1, (b) Smad2 and (c) GAPDH. The original images of Fig. 3e were (d) Shh, (e) Smo and (f) GAPDH. The original images of Fig. 3f were (g) α-SMA, (h) fibronectin and (f) GAPDH.

Supplementary Fig. S4. The original image Fig. 4g, h. The original images of Fig. 4g were (a) TGF-β_1, (b) Shh and (c) GAPDH. The original images of Fig. 4h were (d) TGF-β_1, (e) Shh and (f) GAPDH.

Supplementary Fig. S5. The original image of Fig. 5 were (a) TGF-β_1, (b) Shh, (c) Smo, (d) Gli-1, (e) α-SMA, (f) GAPDH, and (g) fibronectin.

Supplementary Fig. S6. The original image of Fig.6. The original images of Fig. 6a were (a) (a) TGF-β_1, (b) fibronectin and (c) GAPDH. The original images of Fig. 6c were (d) Shh, (e) fibronectin and (f) GAPDH.

Supplementary Fig. S7. The original image of Fig.7. The original images of Fig. 7a were (a) TGF-β_1 and (b) GAPDH. The original images of Fig. 7b were (c) Shh and (d) GAPDH. The original images of Fig. 7c were (e) TGF-β_1, (f) α-SMA and (g) GAPDH. The original images of Fig. 7d were (h) Shh, (i) α-SMA and (j) GAPDH.

Supplementary Fig. S1.

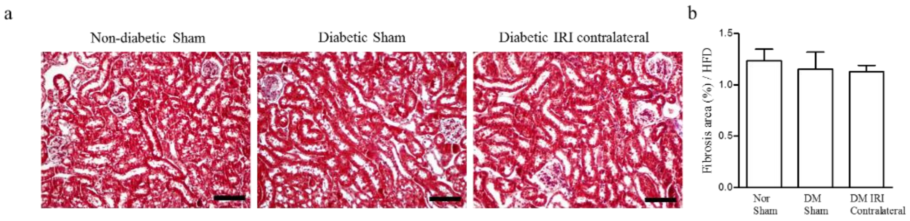

Supplementary Fig. S2.

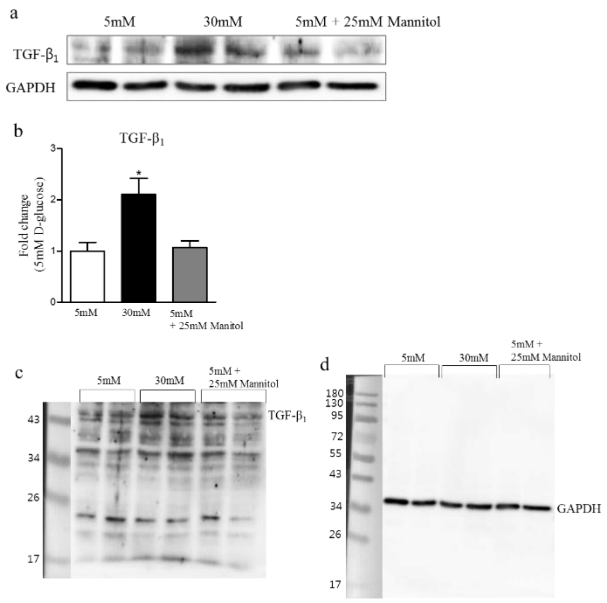

Supplementary Fig. S3. The original image of Fig. 3d-f

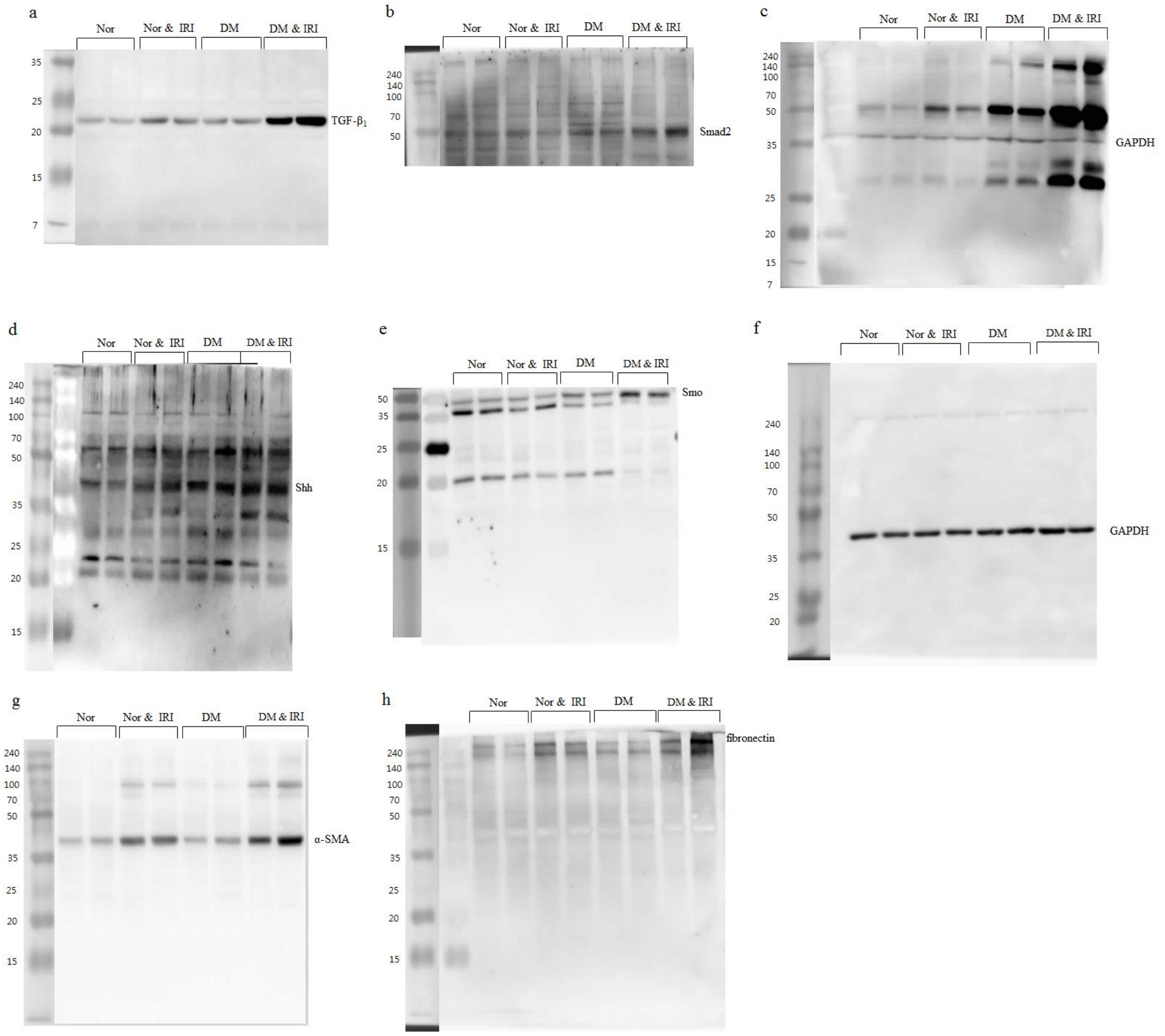

Supplementary Fig. S4. The original image of Fig. 4g, h.

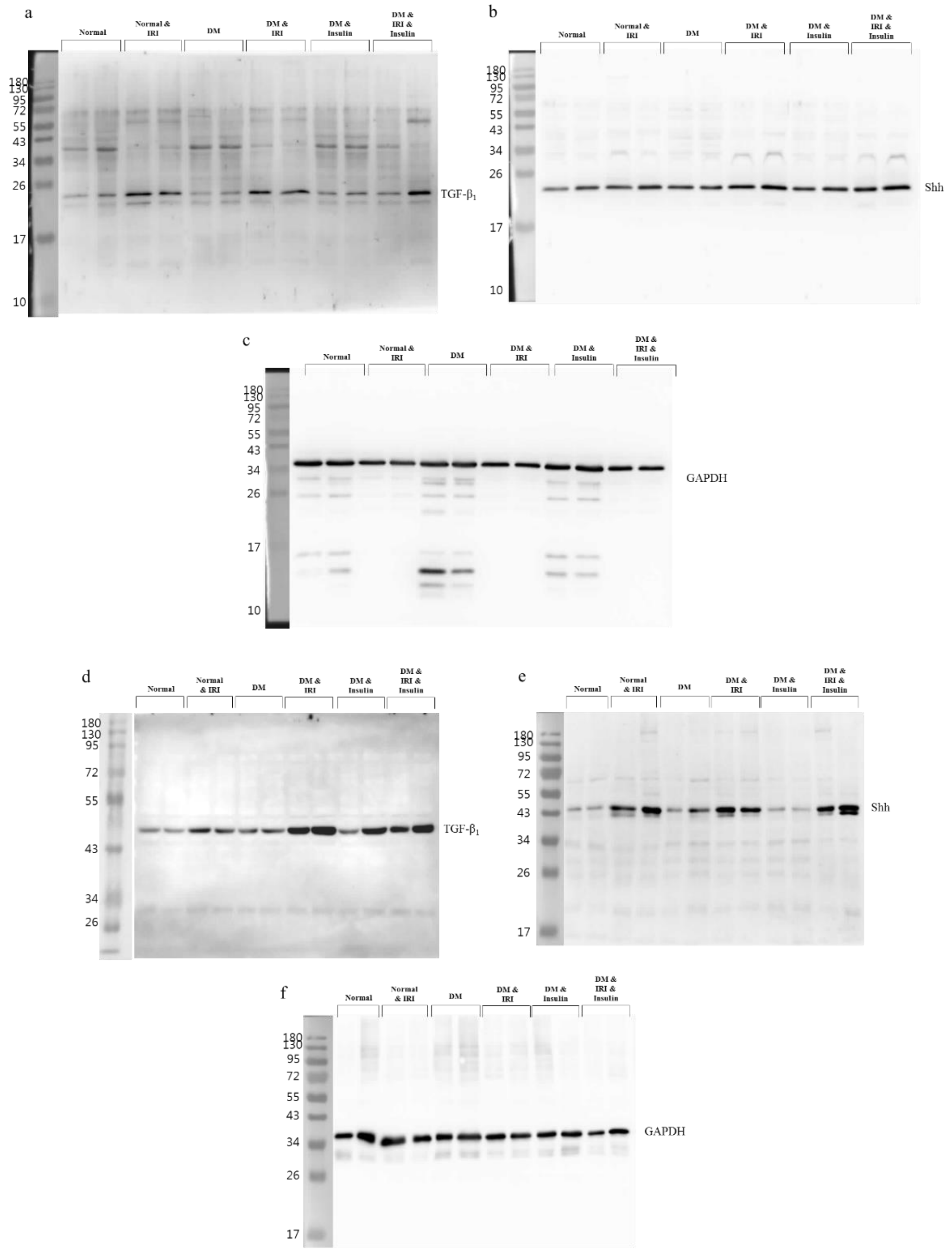

Supplementary Fig. S5. The original image of Fig. 5

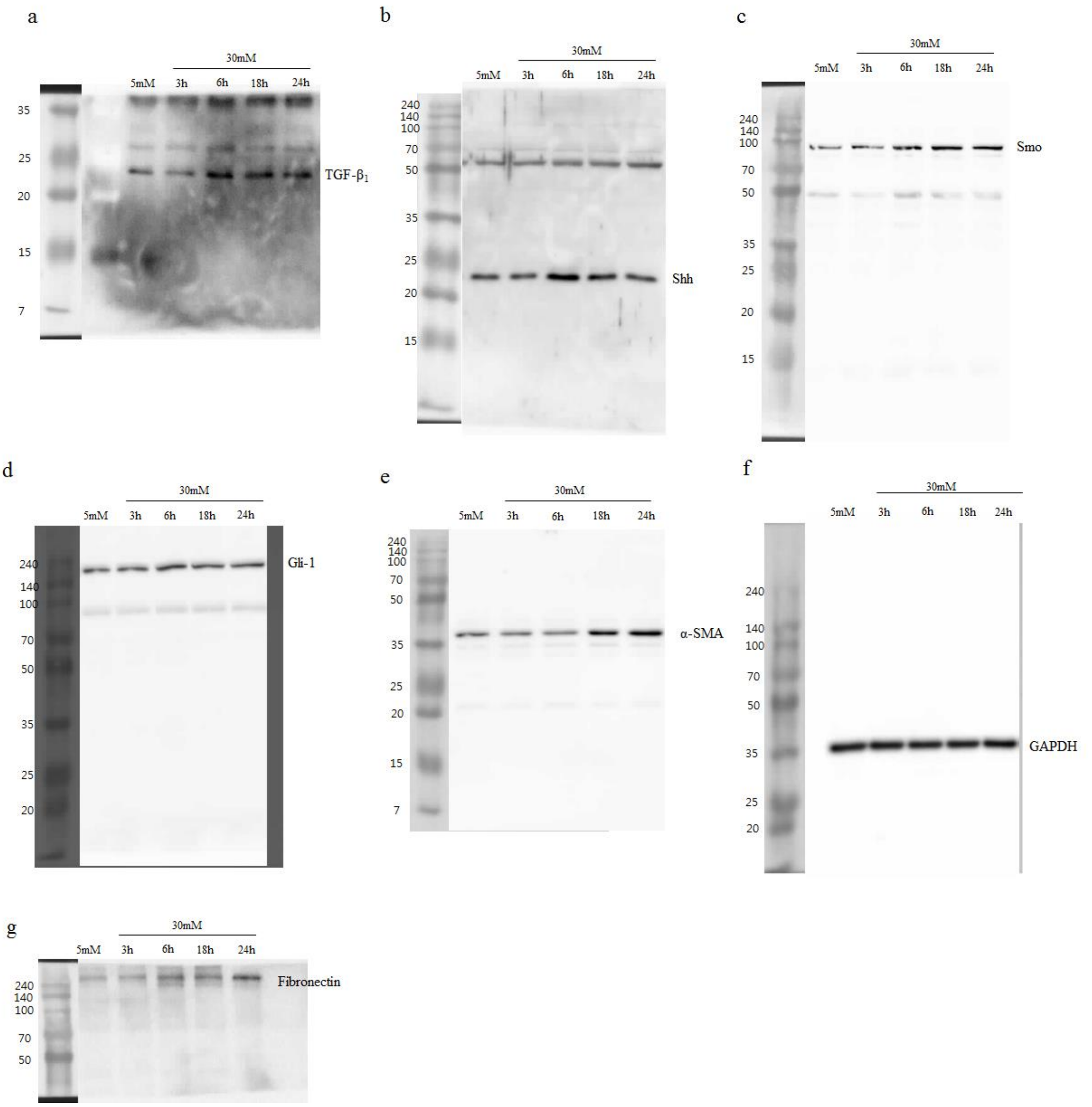

Supplementary Fig. S6. The original image of Fig. 6

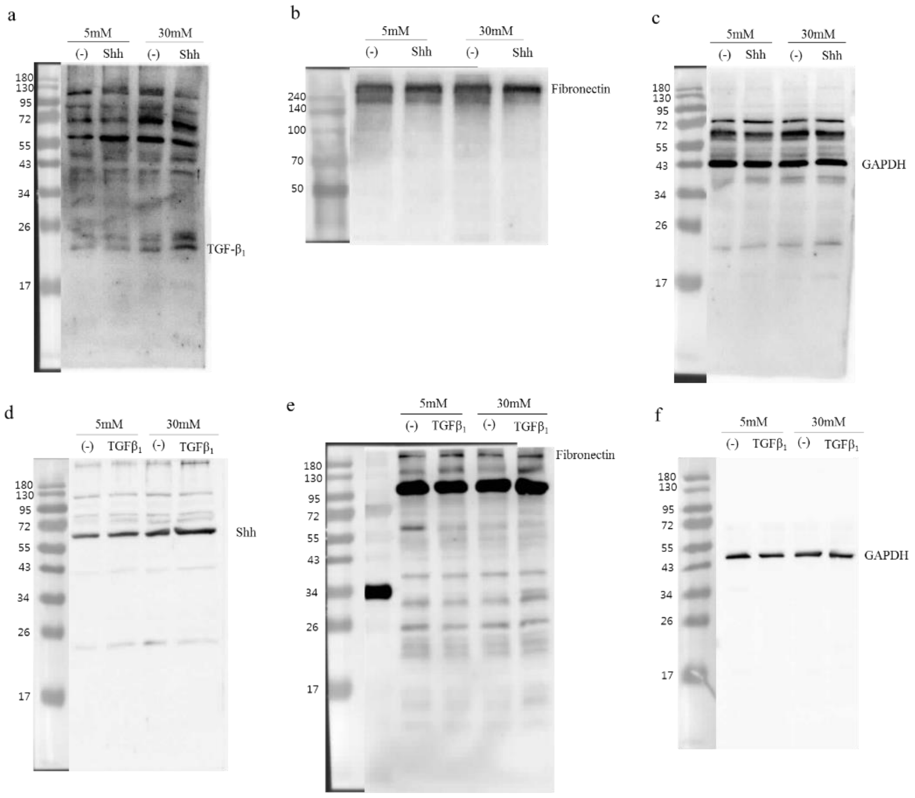

Supplementary Fig. S7. The original image of Fig. 7

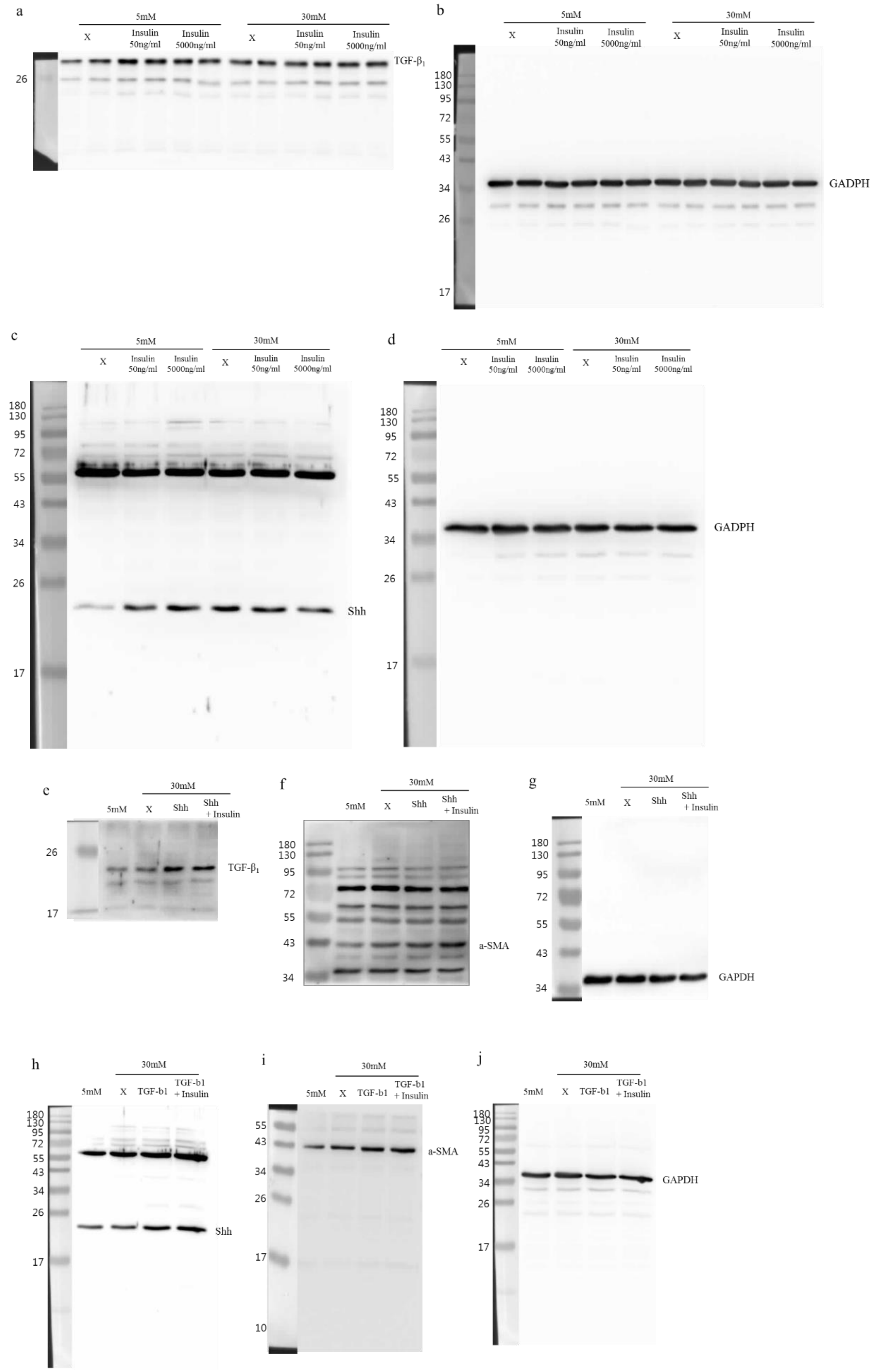

Supplement: Supplementary file 1 — Supplementary Information [file 41598_2017_16977_MOESM1_ESM.pdf]
